# Crocodilian Nest in a Late Cretaceous Sauropod Hatchery from the Type Lameta Ghat Locality, Jabalpur, India

**DOI:** 10.1371/journal.pone.0144369

**Published:** 2015-12-07

**Authors:** Rahul Srivastava, Rajeev Patnaik, U. K. Shukla, Ashok Sahni

**Affiliations:** 1 361/II, Tikait Rai LDA Colony, Lucknow-226017, India; 2 Centre of Advanced Study in Geology, Panjab University, Chandigarh-160014, India; 3 Department of Geology, Banaras Hindu University, Varanasi-221006, India; University of Oxford, UNITED KINGDOM

## Abstract

The well-known Late Cretaceous Lameta Ghat locality (Jabalpur, India) provides a window of opportunity to study a large stable, near shore sandy beach, which was widely used by sauropod dinosaurs as a hatchery. In this paper, we revisit the eggs and eggshell fragments previously assigned to lizards from this locality and reassign them to crocodylomorphs. Several features point to a crocodilian affinity, including a subspherical to ellipsoidal shape, smooth, uneven external surface, discrete trapezoid shaped shell units with wide top and narrow base, basal knobs and wedge shaped crystallites showing typical inverted triangular extinction under crossed nicols. The crocodylomorph eggshell material presented in this paper adds to the skeletal data of these most probably Cretaceous-Eocene dryosaurid crocodiles.

## Introduction

The Late Cretaceous Lameta Formation of India is widespread and contains a great wealth of sauropod dinosaur egg nests. From this predominantly sauropod dinosaur hatchery, we report for the first time a crocodile egg nest. In a global context, the fossilized crocodile nests are very rare (around 20 reported till date) [1] and our find implies that this sauropod hatchery of Lameta Formation was also occupied by other large reptiles like crocodiles also for laying eggs. This paper is a sequel to an earlier one [2] wherein a purported lizard nest was described from the Maastrichtian Type Lameta Ghat section, near Jabalpur. Additional work now shows that attribution of the eggshells to a large lizard was incorrect. Instead, examination of ultrastructure of the eggshell indicates the nest belongs to a crocodile. The identification of eggs as those of a lizard in last communication [2], was primarily based on the size of these eggs and their amorphous (non-crystalline) structure as seen in radial and cross sections. However, more analysis and comparison with the recent and fossil crocodilian eggshells reveals that the eggshells belong to crocodylomorphs. These crocodiles most probably belong to a Cretaceous-Eocene group of dryosaurid crocodiles that were dominant in South Asia during that time range and survived the K-Pg boundary.

In the previous paper [2], an error was made while making the measurements. The size of the eggs that was given in the previous paper was apparent and we did not consider the tangential erosion and sectioning of the outcrop. Another error in identification was that we chose a highly silicified portion of an eggshell for our thin section study. Based on the revised comparisons, we conclude here that the eggshell belongs to crocodylomorphs, closest to that described from Crocodylia [3, 4]. The dominant crocodiles in the drifting Indian Plate in the Cretaceous and early Paleogene were the mesoeucrocodiles [5–11], therefore there is a possibility that the nest belongs to this group. However there is no direct physical evidence for this.

**Fig 1:**
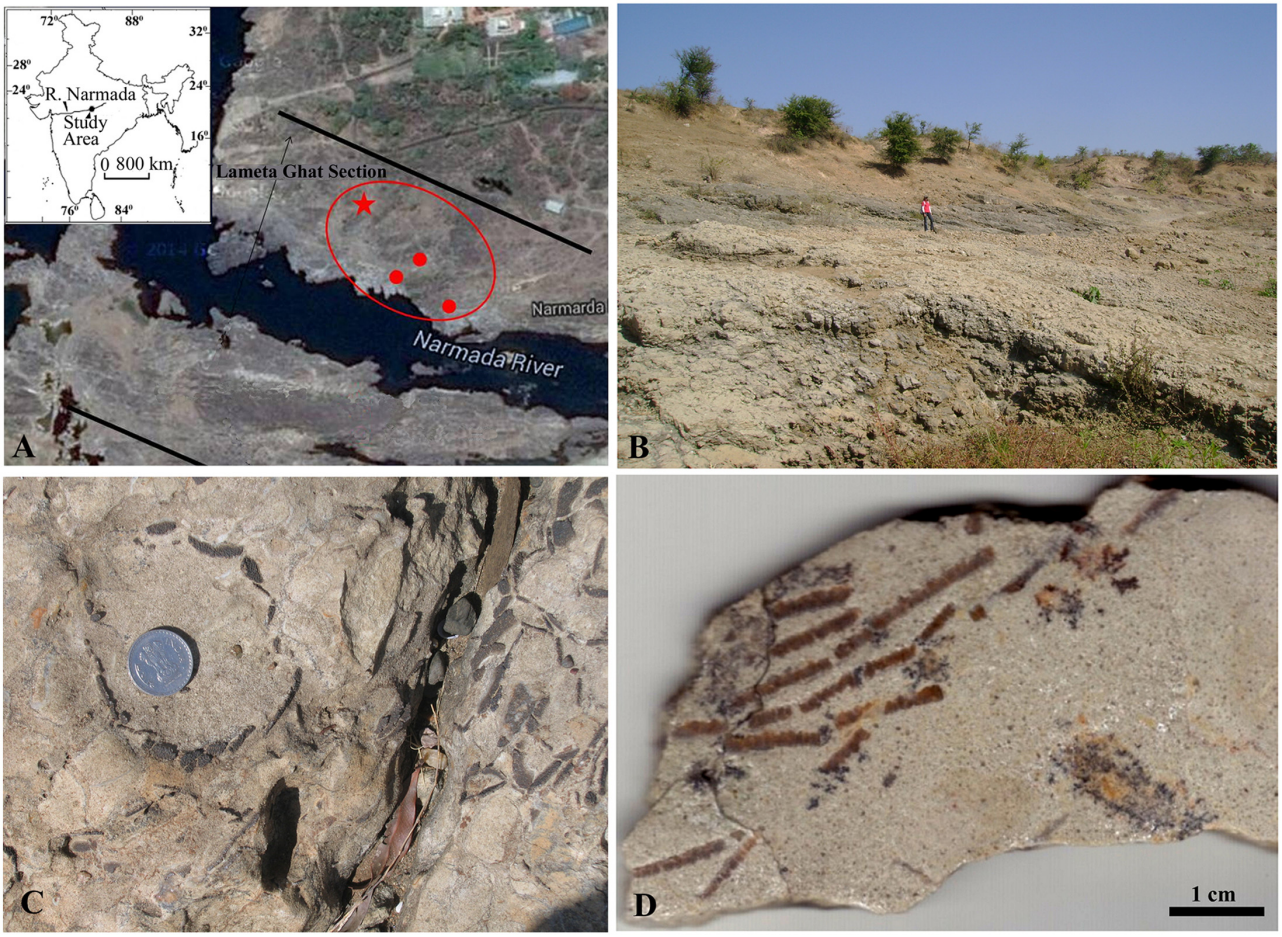
Showing the study area, location of crocodile, dinosaur egg nests and a polished eggshell section. **A.** The study area (inset) and the location of crocodile (red star) and sauropod (red dots) nesting sites at Lameta Ghat (modified from Google Earth). **B.** Location of crocodile and dinosaur eggs, northern bank of Narmada River. **C**. Dinosaur nest of *Megaloolithus jabulpurensis* found in association with the crocodile nest at Lameta Ghat. **D.** Polished radial sections of *Megaloolithus* eggshell fragments in concentric arrangement (VPL/CCE - 5C).

In a spatial context, the crocodylomorph nest lies within the range from where sauropod nests have been reported before [12] and herein (Fig 1A). The concentric layered arrangement of these sauropod eggshells (Fig 1C and 1D) and their shell microstructure confirms that the eggshells belong to those of *Megaloolithus jabulpurensis* [13].

In spite of the concerted efforts of several group of workers, the palaeo-depositional environments of the Lameta in the Type area of Jabalpur and in the surrounding outcrops, has been a matter of debate [2, 12–18]. Shukla and Srivastava [2] proposed the presence of an alkaline lagoon at the Lameta Ghat locality while others have argued for the calcareous limestone to be pedogenic in origin. Fossils found within the limestone have consistently shown a freshwater character [17, 19–22]. The only estuarine element recorded is the ray *Igdabatis* [23]. It is more than possible that during the deposition of the Lameta, the shoreline was at any one point of time not far away from the coastal fluvial complex that the sedimentation suggests. Keller et al. [24] have demonstrated the presence of a Trans Deccan Seaway traversing peninsular India during the Late Cretaceous—early Paleogene during the eruption of the Deccan volcanic.

## Material and Methods

**Fig 2:**
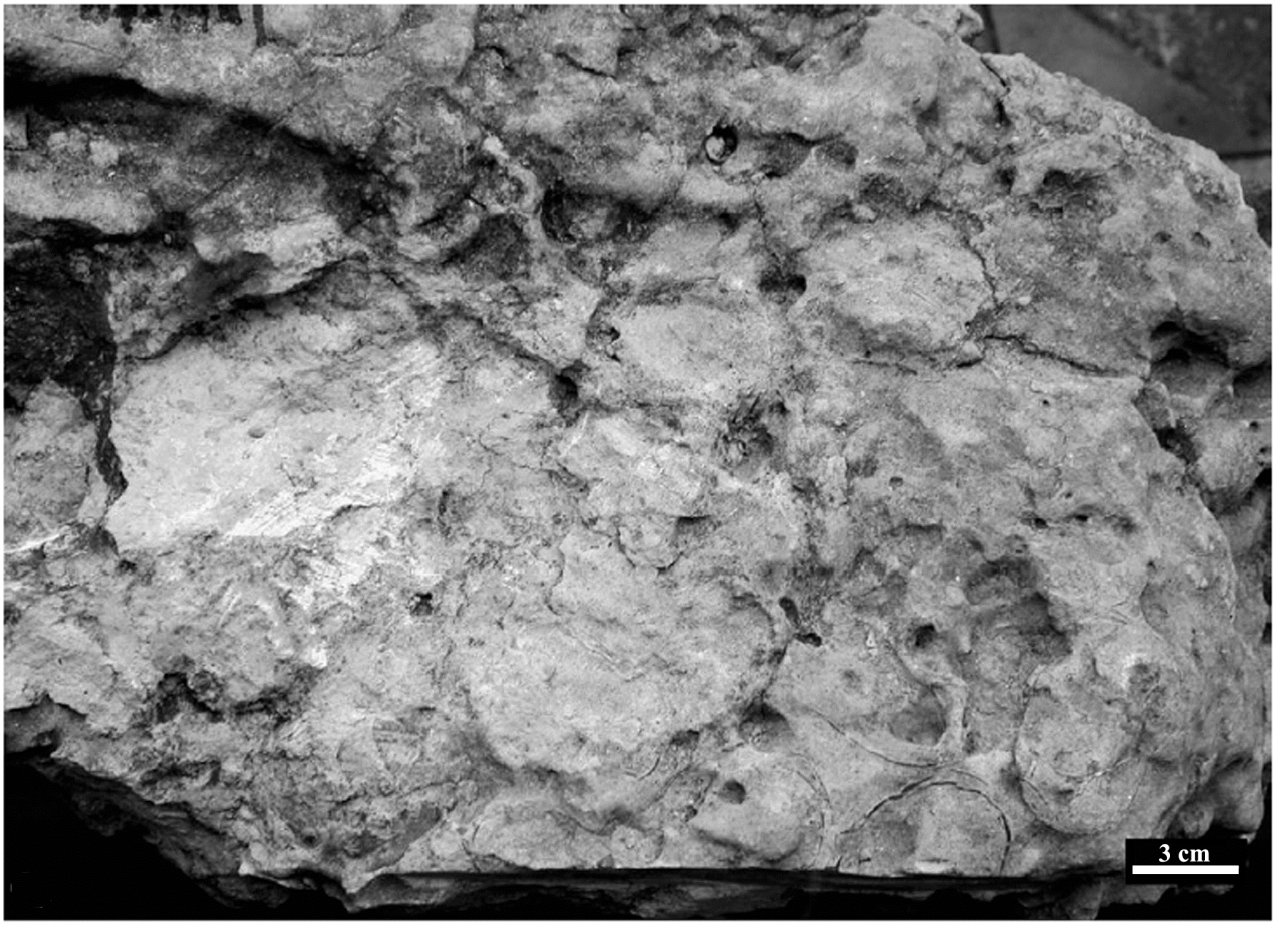
Photograph showing nest with eggs (LU/RS 01/9).

Shukla and Srivastava [2] recovered a well-preserved nest with nine partial and two complete eggs from Lameta Ghat, (Fig 2). This eggshell material (catalogue no. LU/RS-01/9) described by Shukla and Srivastava as lizard eggshell [2] is catalogued in the Department of Geology, Lucknow University, Lucknow, India. Eggshells from their collection (VPL/CCE 3–5) were radially sectioned and polished and viewed under the light microscope Leica S8 APO and Leica SM 2500. These were then photographed under the crossed nicols using petrographic microscope Olympus CX31. The polished sections were then etched using 5% formic acid for 5 seconds and viewed under the scanning electron microscope (JEOL 6940). Eggshell (VPL/CCE- 1 & 2) outer, inner and freshly broken radial surfaces were also viewed under the SEM. The electron microscope and light microscope material used in this study is catalogued in the CAS in Geology, Panjab University Chandigarh, India.

Both the Universities (Panjab University and Lucknow University) have long and established museum repositories with full access of materials to all scientists and can be visited freely without permit at any time. There is a curator and staff dedicated to repository section.

## Description

**Fig 3:**
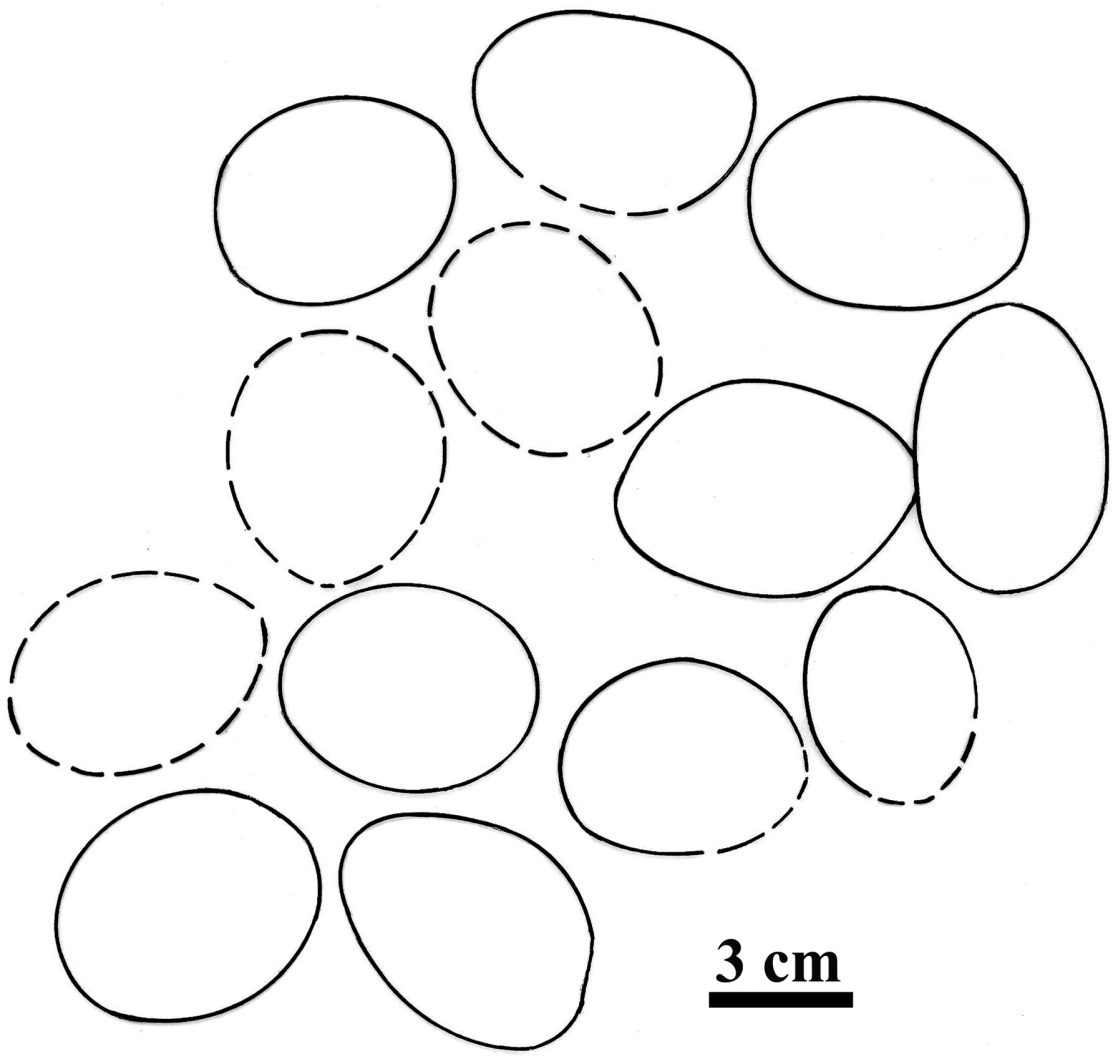
A schematic diagram showing position of eggs in the nest. Incomplete eggs shown by dotted lines.

**Fig 4:**
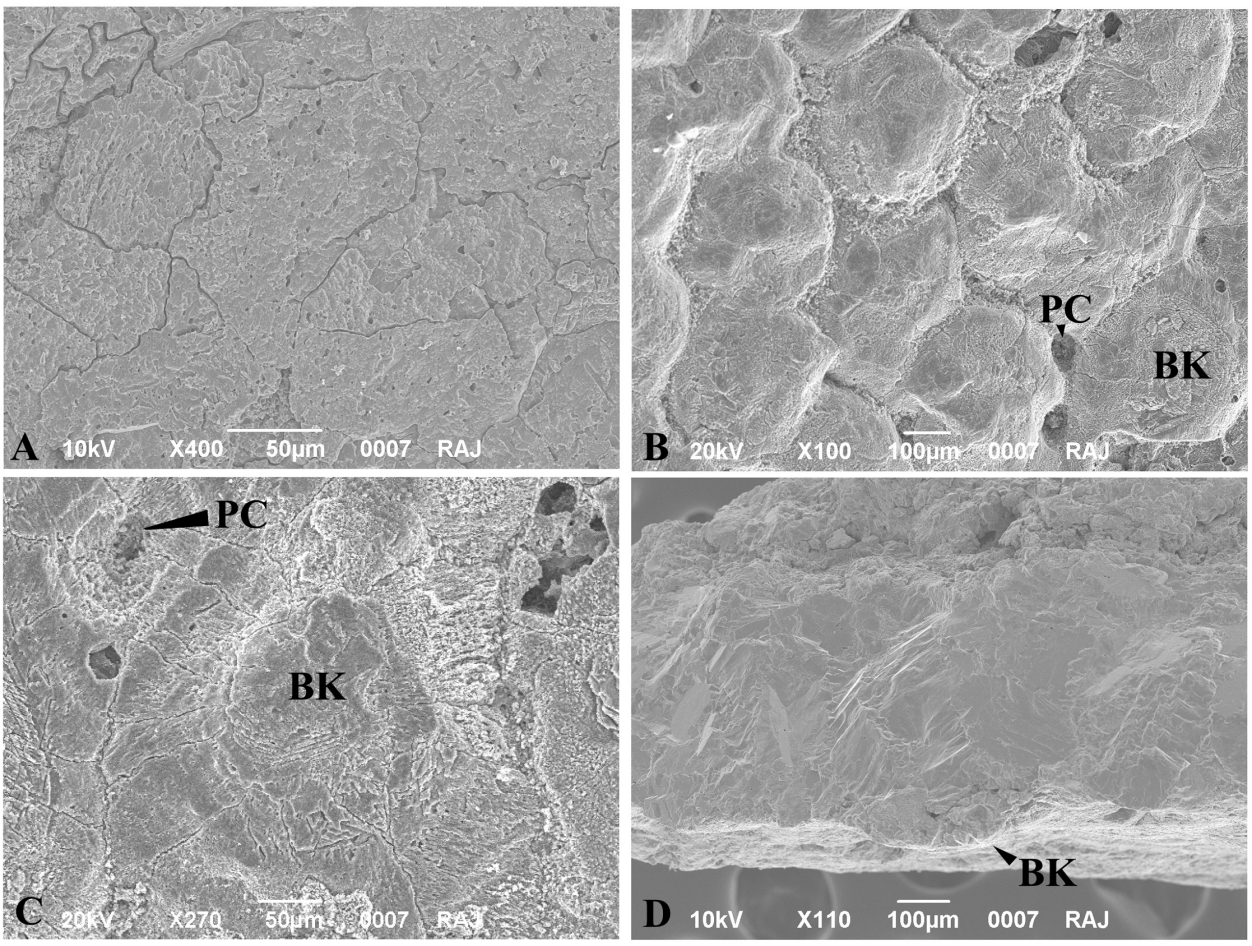
Showing the crocodylomorph eggshell outer, inner and radial surfaces. **A.** Crocodylomorph eggshell (VPL/CCE 1–2) outer surface. **B.** Inner surface showing basal knobs (BK) and pore canal openings (PC). **C.** A portion of inner surface enlarged to show wedges radiating away from the basal knobs. **D.** A freshly broken radial surface showing trapezoid shaped shell unit with wedges radiating away from the basal knobs.

**Fig 5:**
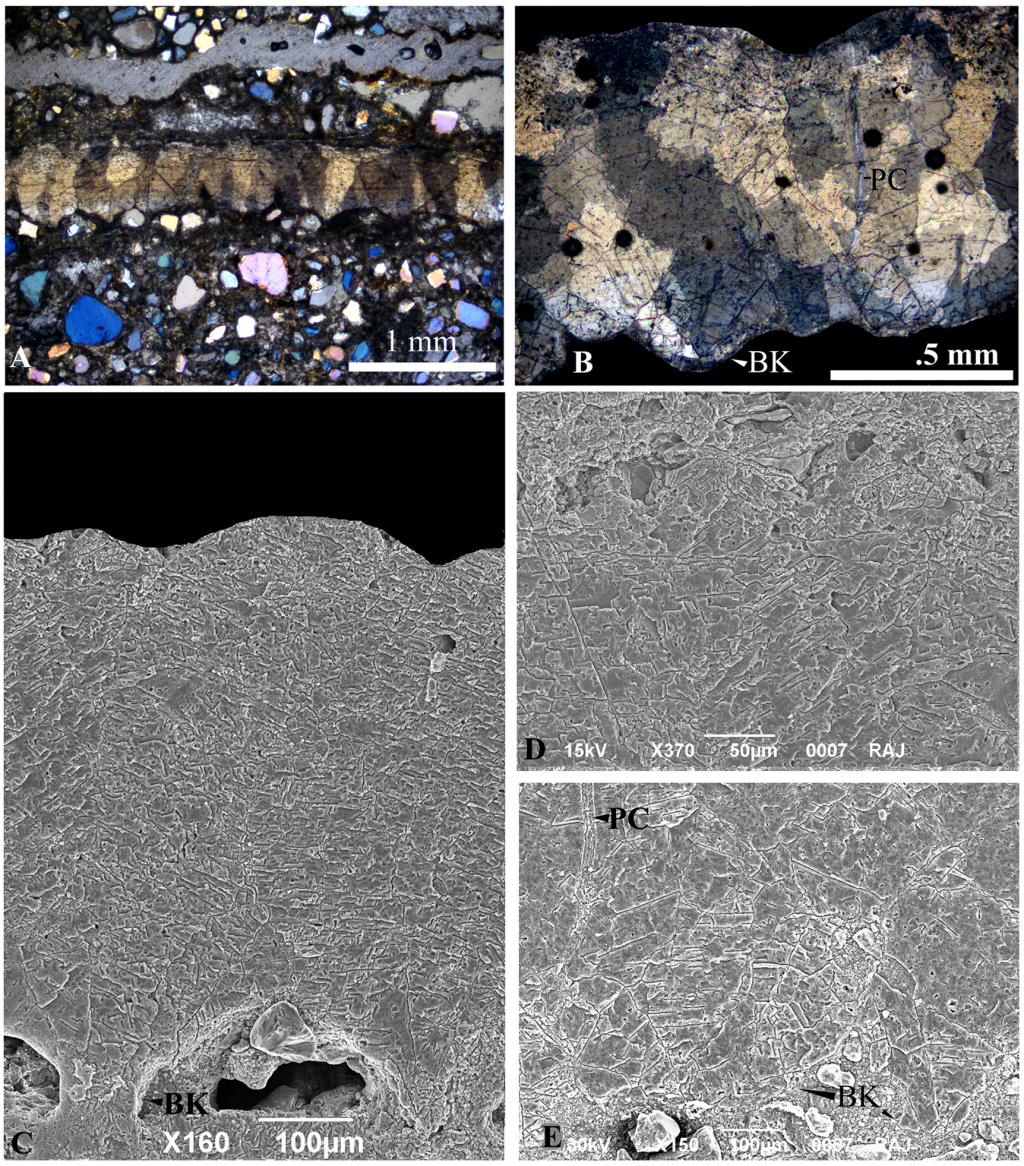
Crocodylomorph eggshell sections under petrographic and scanning electron microscopes. **A.** Crocodylomorph eggshell (VPL/CCE 3) section under crossed nicols showing floating quartz grain in calcareous matrix and horizontal accretion lines and the typical crocodilian inverted triangle extinction pattern. **B.** One portion of the eggshell (VPL/CCE 4) cut tangentially to show overlapping trapezoid shell units and irregular blocky extinction. **C.** Scanning Electron Micrograph of polished and etched eggshell section showing basal knob (BK) and smooth but uneven outer surface. **D.** The outer part of the same section enlarged showing herringbone pattern. **E.** inner layer showing wedges diverging outwards from the basal knobs (BK) and the pore canal (PC).

**Fig 6:**
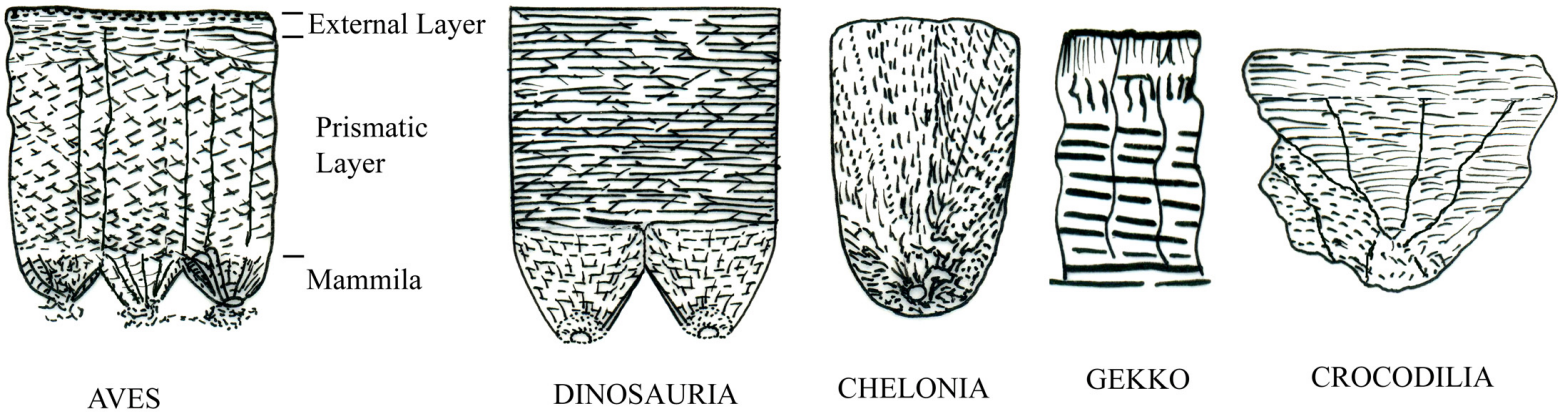
Line drawing based on published papers showing basic structure of shell units found in birds, dinosaurs, turtles, lizards and crocodiles (not to scale).

**Fig 7:**
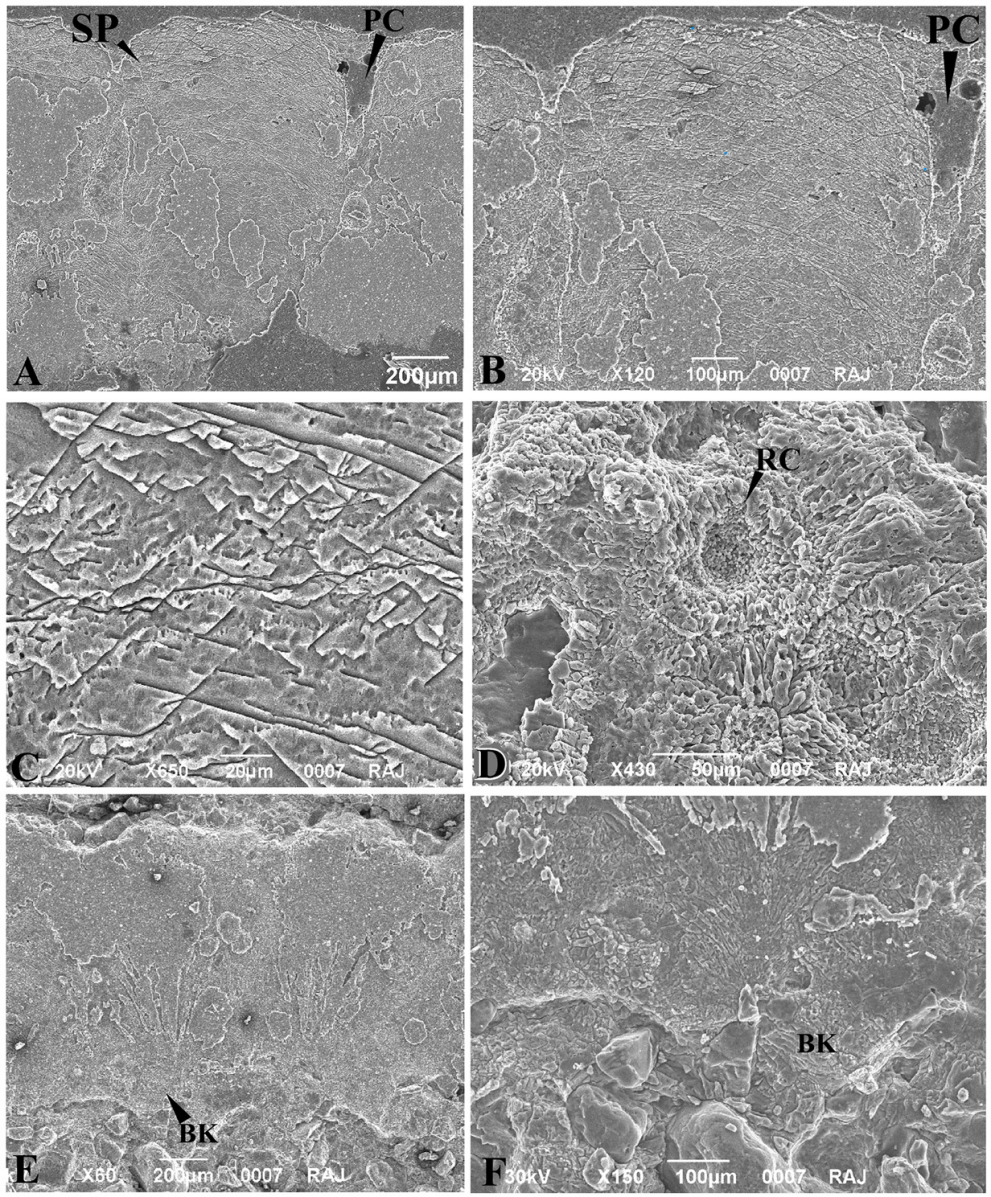
Comparison of the present eggshells with those a Pliocene Siwalik crocodile and dinosaur eggs of *Megaloolithus jabulpurensis* under crossed nicols. A, The present eggshell thin section showing crocodilian features described above. B, Section of the fossil **c**rocodylian eggshell (VPL/RP_RE-2) collected from the Siwaliks [41] exhibiting the characteristic horizontal accretion lines (HAL) and inverted triangle extinction (ITE) pattern. C, D and E, *Megaloolithus jabulpurensis* (VPL/CCE - 5C) showing fan shaped spheruliths, basal knobs (BK) sweeping extinction, arcuate accretion lines (AAL) and tuberculate outer surface. E, one of the spherolith further magnified.

The eggs in the nest are in general sub spherical to ellipsoidal in shape with their size being ~ 68 x 44 mm (elongation index 1.54) (Figs 2 and 3). The thickness of the eggshell ranges between 0.43–0.47 mm [2]. The external surface is uneven (somewhat undulating) and smooth (does not show corrosion or contain nodes or tubercles) (Fig 4A) but the inner surface shows packing of basal knobs and pore canal openings (Fig 4B). The discrete shell units are trapezoidal in shape, the top is wider and the base becomes gradually narrower and bears the basal knobs (Figs 4D, 5A–5C and 6). These discrete units are attached to each other laterally and are marked by straight pore canals that pass through the entire shell (Fig 5B and 5E). Under the crossed nicols inverted triangular extinction wedges are distinct (Figs 5A and 5B and 7B). Irregular extinction pattern, criss-cross calcite cleavages disrupting horizontal accretion patterns at places indicate recrystallization of the eggshells. The space between the basal knobs is less compared to those seen in extant crocodiles, for example in *Caiman latirostris*, *Gavialis gangeticus* and *Crocodylus porosus* [4, 1]. The innermost layer is characterized by radiating wedges originating at the basal knobs (Fig 5C & 5E). The horizontal accretion (growth) lines or the laminated tabular structure typical of crocodylomorph eggshell [4, 25, 26, 27] is visible throughout the shell thickness (Fig 5A). The middle and the outer portions of the eggshells show blocky texture with both discontinuous oblique and tabular cleavage pattern (Fig 5D). However, the three distinct layers found in extant crocodile eggshells are not observed in these eggshells.

## Comparisons

The present eggshells are clearly distinct from those of the lizards in lacking the characteristic numerous, slender densely packed columnar shell units [28, 26, 29] (Fig 6). Bird eggshells would differ from the present eggshells in having the typical three layered (mammillary, prismatic and external) structure [28, 30]. Eggshell of turtles are distinct from the present eggshells in having the cylindrical shell units spherulite with spherical base made up of aragonite crystallites [28, 30]. Dinosaurs on the other hand have spheroliths as the shell-forming unit. Sauropod dinosaurs in particular show sweeping extinction pattern under crossed nicols [28].

**Fig 8:**
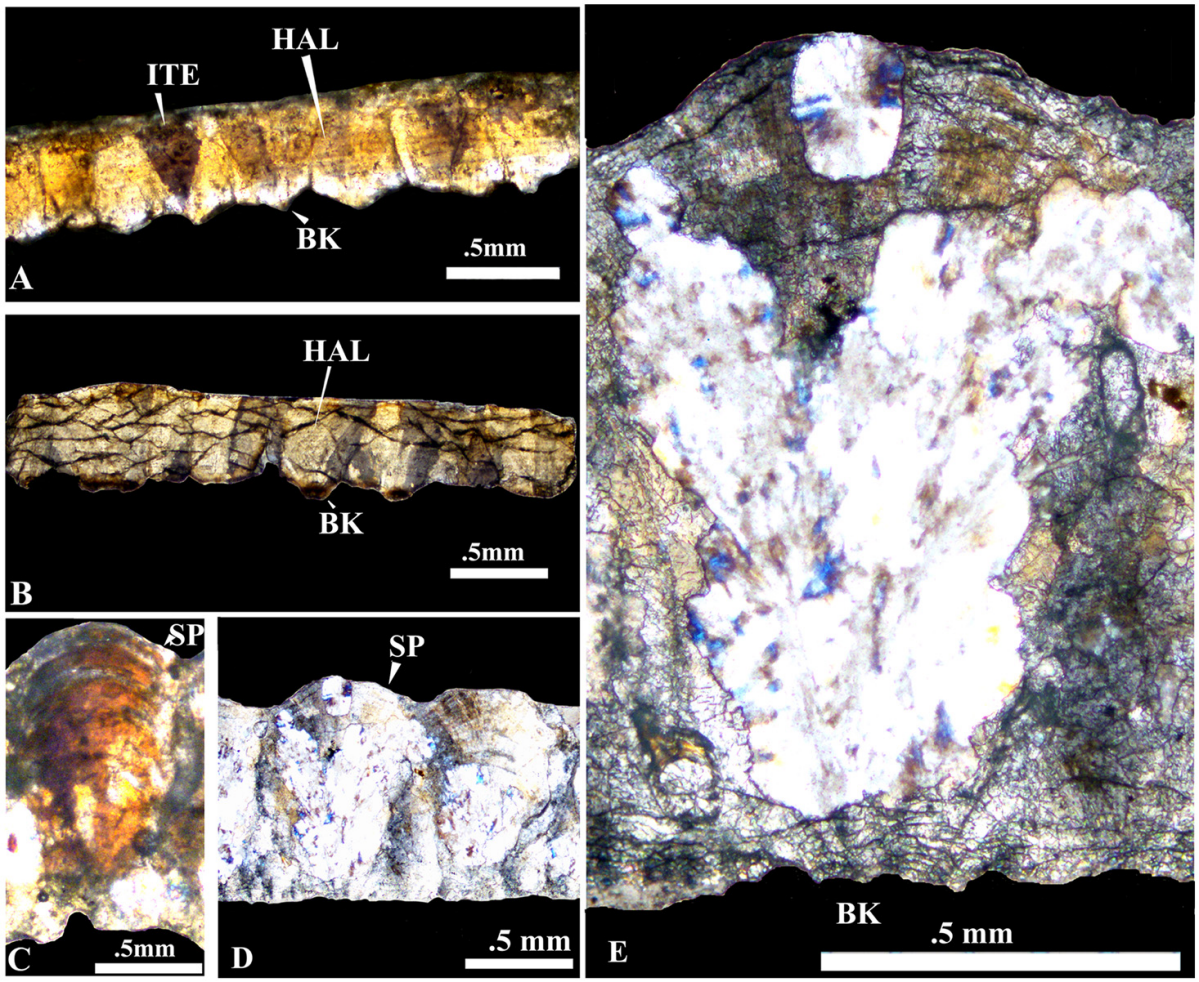
Scanning Electron Micrographs of *Megaloolithus jabulpurensis* (VPL/CCE - 5C) eggshells. A, Fan shaped spherolith (SP) and pore canal (PC). B, Spongy layer of the spherolith magnified showing arcuate accretion lines and various fracture patterns.C, Spongy layer further magnified to show the typical “Herring-Bone” Pattern. D, Inner eggshell surface showing mamillae with resorption crater (RC). E, a pair of partly silicified fan-shaped speroliths with basal knobs. F, Basal knob (BK) magnified to show radiating patterns.

Compared to the present eggs and eggshells those of the dinosaur *Megaloolithus jabulpurensis* eggs are circular to sub-circular, much larger (~ 14 cm in diameter, Fig 1C) and thicker (~1.5 mm, Fig 7C), have a tuberculated external surface, an inner surface with mammillae showing resorption craters (Fig 8D), fan-shaped spheroliths showing arcuate accretion lines, radiating fracture patterns and sweeping extinction pattern under crossed nicols (Fig 7C) [13]. Fossilized crocodilian eggshells from Pliocene sediments of Siwaliks [31] are very similar to the present material in having a smooth outer surface, prominent basal knobs, trapezoid shell units, horizontal accretion lines, and blocky extinction pattern under crossed nicols (Fig 7A).

The egg size (~ 68 x ~44 mm), elongation index (IE) (~1.54) and thickness (~0.5 mm) fall closest to the size (66 x 46 mm and 60 x 40.9 mm), IE (1.43 and 1.54) of modern alligatorid *Caiman latirostris* [4, 1,32]. Egg size, EI and shell thickness of modern gavialid *Gavialis gangeticus* is around 82 x 56 mm; 1.46; .30-.59 mm respectively and that of crocodylid *Crocodylus porosus* is ~77 x 52 mm; 1.48; .53 mm, respectively [3]. *Caiman latirostris* eggshells surface is corroded, whereas that of *Crocodylus porosus* is smooth with stepped concentric ring structure on the rim of the pore openings [4]. The outer surface of *Gavialis gangeticus* is generally smooth with funnel-like pores [4, 25]. Unlike the present eggshells all these extant crocodiles show wide inter- basal knob space/cavity that reaches almost half the width of the eggshell [3]. Among the fossil crocodiles described so far the present eggs come closest in size and elongation index (size = 68 x 44 mm; EI = 1.55) to those from the Eocene Bridger Formation, USA reported by Hirsch and Kohring [33, 1]. However, the Eocene eggshells are thicker (0.75 mm) than the present eggshells. The fossil crocodilian eggshells reported from Late Cretaceous intertrappean beds, Bombay [34], differ from the present eggshells in being thinner (~.35 mm) showing distinct mammillary layer and with diverging micro crystallites. The Pliocene Siwalik crocodile eggshells [31] are thinner, have smooth outer surface, discrete wedges, mammillary layer or inner layer with diverging crystallites and distinct continuous tabular accretion lines (Fig 7B). Another crocodilian egg from the Siwaliks of Pakistan [3] differs from the present egg and eggshells in being larger in size (84 x 64 x 54 mm) and having an ornamented outer surface.

## Discussion

The microstructure of fossil and extant crocodilian eggshells has long been debated. Ferguson [35] found five separate layers in the *Alligator mississippiensis* eggshell. However, Hirsch [25] considered the presence of a single calcified layer in fossil crocodiles, but found the basal plate groups, the mammillary layer of Ferguson [35] and the wedge-like crystals as separate entities. Hirsch [25] followed by Mikhailov [28, 26] consider the crocodyloid morphotype as single-layered eggshell with ‘rough’ shell units. Kohring and Hirsch [36] following this approach established the Krokolithidae oofamily, which has been used by other authors [3, 37, 38]. However, recent studies [39, 40, 1] on both fossil and recent crocodiles have shown that crocodilian eggshell is composed of several structural layers attesting to the observation of Ferguson [35]. A typical feature of crocodilian eggshell thin section under crossed nicols is the presence of blocky extinction with an upside down triangular shape [1].

Crocodylomorphs are characterized by the presence of ellipsoidal or elongated eggs, mostly ornamented egg surface with circular, step-like concentric erosion pore openings [1]. The sub-spherical to ellipsoidal shape and size (~68 x 44 mm) of the present eggs fall within the range of those of the extant and extinct crocodylomorphs. More importantly, the present eggshells show discrete wedge-like shell units, the typical crocodylomorph blocky inverted triangle exinctions and non-branching pore canals. Though, the present egg shells do not show the three layers of extant crocodiles, the horizontal tabular accretion feature characteristic of all the crocodylomorphs is present.

The identity of the producer of fossil eggs is best corroborated by finds of embryos within the eggs or by associated hatchlings. Such is not the case here and therefore there is no physical evidence to assign the crocodilian nest to a specific crocodylomorph group. However, crocodile elements mainly isolated teeth and vertebrae including a few fragmentary jaws are known from several sedimentary horizons associated with the Deccan Volcanics. In such a case it is necessary to evaluate the possible identity of the producer by an analysis of the crocodiles that inhabited the Indian Plate during the Late Cretaceous. There is only one taxon, *Pabwehshi pakistanensis*, from the Late Cretaceous Pab Sandstone of Pakistan, that has been definitely assigned to the Mesoeucrocodilia [5]. The authors suggest that the Pakistani genus is a baurusuchid related closely to forms earlier described from Brazil and Argentina. Stratigraphically the Pab Sandstone represents a marginal facies of the Indian Plate suggesting a delta complex with basinwards, shelf and slope deposits. The Pab Sandstone is sourced from sediments derived from the Deccan Volcanics and coeval rocks. From a phylogeny point of view, Buscalioni et al [41] presented a global distribution of Late Cretaceous crocodylomorphs, but he could not place the isolated fragmentary crcodylomorph data from the Late Cretaceous of India in his distribution map, primarily because the data is represented mainly by the isolated teeth [11, 42, 43]. The mesoeucrocodilians found on the Indian Plate (Pakistan and Indian localities) may be of intermediate nature and indicate close affinities with those found in Madagascar, South America [44, 45, 46] and Africa [47, 48, 49, 50].

The depositional palaeoenvironments of the dinosaur nest-bearing Lameta facies have been discussed at length by several workers [12, 14–16] including the specific locality mentioned in this paper [2, 24]. There is a general consensus that the nest-bearing levels are pedogenic in nature. In the present instance, the find of a crocodilian nest along with the nests of large sauropod dinosaurs specifically suggests a more nearshore, lagoonal facies within a stable sandy beach depositional setup [2]. In this environment as well, pedogenesis occurs suggesting stability of the supratidal deposits and provides some clues to the egg-laying behavior of titanosaurid dinosaurs [12]. This further adds to our knowledge of hatchery strategies of early crocodylomorphs belonging most probably to the South Asian dryosaurids, that go on to survive the Cretaceous-Pg extinction event.

## Conclusions

Crocodile eggshells have been reported from several localities throughout the world and are well known from India as well from the K/Pge transition beds of the Mumbai Intertrappeans to Siwalik occurrences [3, 34, 31]. There are excellent studies of the shell ultrastructure of several taxa of modern crocodiles including the *Caiman*, *Alligator* and the more common *Crocodylus*. Additional work on the Lameta crocodile nest confirms that it does not belong to the varanoid lizards as previously indicated. Detailed ultrastructure of the eggshell and the tabular crystal structure of the mammillae, the thickness and radial structure suggest crocodilian affinities. Presence of ellipsoidal eggs, trapezoid shaped wedge like discrete shell units with blocky extinction pattern places the present eggs and eggshells in Crocodylomorpha. The dominant crocodilian in the Cretaceous and early Paleogene of India belong to the mesoeucrocodiles, family Dryosauridae.

Buscalioni et al. [41] in a fairly comprehensive review have tried to trace the evolutionary phylogeny of the crocodylomorphs spatially as well as temporally. Relevant here is the fact that they consider the crocodylomorphs to be monophyletic. The basal group comprises the mesoeucrocodilians which are well represented in the Indian plate even though the absence of skulls and other distinguishing elements, make it difficult at this stage to precisely designate the taxa involved or their affinities because of the isolated material. With a few exceptions, e.g. *Pabwehshi* from the Late Cretaceous Pab Sandstone of Pakistan [5], the Indian material consists either of isolated teeth [42,43] or of isolated vertebrae [11]. Lower jaws have also been described from Dindori, but these are fragmentary and cannot be assigned to a specific taxon [5]. Crocodilian teeth found at Naskal are distinctly serrated and suggest affinities to the basal Ziphosuchia.
