# Correction: Crocodilian Nest in a Late Cretaceous Sauropod Hatchery from the Type Lameta Ghat Locality, Jabalpur, India

**DOI:** 10.1371/journal.pone.0146736

**Published:** 2016-01-05

**Authors:** Rahul Srivastava, Rajeev Patnaik, U. K. Shukla, Ashok Sahni

The captions for Figs 7 and 8 are incorrectly switched. Please see the correct captions here.

**Fig 7:**
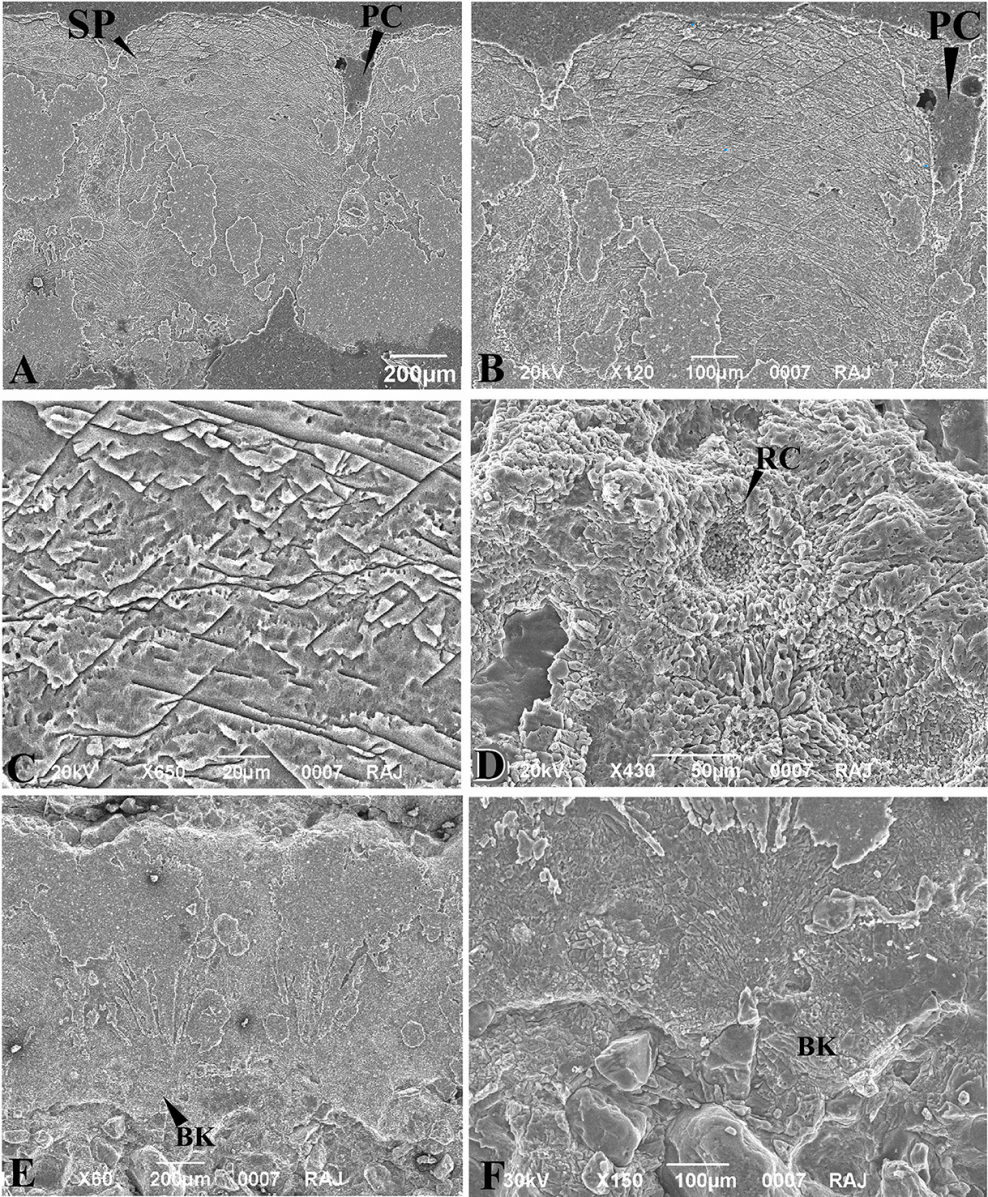
Scanning Electron Micrographs of *Megaloolithus jabulpurensis* (VPL/CCE—5C) eggshells. A, Fan shaped spherolith (SP) and pore canal (PC). B, Spongy layer of the spherolith magnified showing arcuate accretion lines and various fracture patterns. C, Spongy layer further magnified to show the typical “Herring-Bone” Pattern. D, Inner eggshell surface showing mamillae with resorption crater (RC). E, a pair of partly silicified fan-shaped speroliths with basal knobs. F, Basal knob (BK) magnified to show radiating patterns.

**Fig 8:**
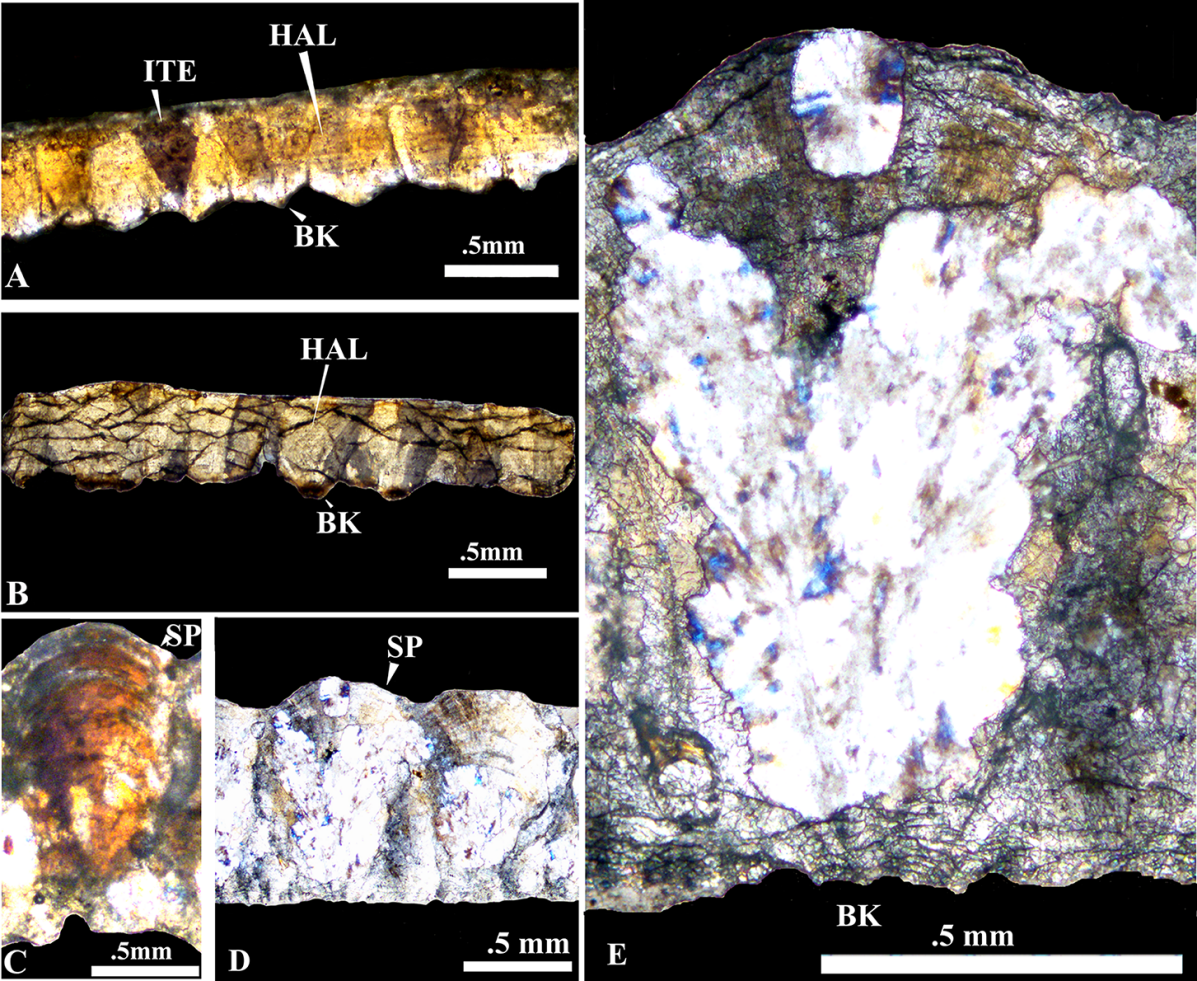
Comparison of the present eggshells with those a Pliocene Siwalik crocodile and dinosaur eggs of *Megaloolithus jabulpurensis* under crossed nicols. A, The present eggshell thin section showing crocodilian features described above. B, Section of the fossil crocodylian eggshell (VPL/RP_RE-2) collected from the Siwaliks [41] exhibiting the characteristic horizontal accretion lines (HAL) and inverted triangle extinction (ITE) pattern. C, D and E, *Megaloolithus jabulpurensis* (VPL/CCE—5C) showing fan shaped spheruliths, basal knobs (BK) sweeping extinction, arcuate accretion lines (AAL) and tuberculate outer surface. E, one of the spherolith further magnified.
